# The putative compatible solute-binding protein ProX from *Mycobacterium tuberculosis* H37Rv: biochemical characterization and crystallographic data

**DOI:** 10.1107/S2053230X18003771

**Published:** 2018-03-23

**Authors:** Jian-Hong Zhao, Jiang-Huai Chen, Yong Wang, Zhi-Ping Wang, Yong-Xing He

**Affiliations:** a Institute of Urology, Lanzhou University Second Hospital, Key Laboratory of Gansu Province for Urological Diseases, Clinical Center of Gansu Province for Nephrourology, Lanzhou 730000, People’s Republic of China; b MOE Key Laboratory of Cell Activities and Stress Adaptations, School of Life Sciences, Lanzhou University, Lanzhou 730000, People’s Republic of China

**Keywords:** ProX, ABC transporter, *Mycobacterium tuberculosis*, substrate-binding protein

## Abstract

In *Mycobacterium tuberculosis*, the *proX* gene encodes a putative compatible solute-binding protein (MtProX). However, it was found through sequence alignment that the MtProX protein has very different ligand-binding residues compared with other compatible solute-binding proteins, implying that MtProX may bind to ligands that are as yet uncharacterized. In this work, it was demonstrated that MtProX binds to polyphenols such as phloretin, monoacetylphloroglucinol and 2,4-dihydroxyacetophloroglucinol with dissociation constants between 20 and 70 µ*M*. Crystals of MtProX were obtained using a precipitant consisting of 0.2 *M* NaCl, 0.1 *M* Tris pH 8.5, 25%(*w*/*v*) polyethylene glycol 3350. The crystals diffracted to 2.10 Å resolution and belonged to space group *P*4_3_2_1_2, with unit-cell parameters *a* = *b* = 90.17, *c* = 161.92 Å, α = β = γ = 90.0°. Assuming the presence of two MtProX molecules in the asymmetric unit, the Matthews coefficient was calculated to be 2.74 Å^3^ Da^−1^, which corresponds to a solvent content of 55%.

## Abstract 2

The putative compatible solute-binding protein ProX from *Mycobacterium tuberculosis* was found to bind polyphenols instead of betaine, choline or carnitine. X-ray diffraction data were collected to 2.10 Å resolution.

## 1. Introduction   

Osmoregulation plays an important role in niche adaptation in bacteria, and the most common strategy is to modulate the intracellular concentrations of osmotically active compounds, also known as compatible solutes, through transporters (Holtmann & Bremer, 2004 ▸). The compatible solutes, which include glycine betaine, ectoine, proline *etc.*, are metabolically inert and do not interfere with cell physiology even at very high concentrations (Hoffmann *et al.*, 2008 ▸). A subfamily of ATP-binding cassette transporters (ABC transporters), which are usually multimeric and contain membrane-associated ATPase subunits, have been identified to be involved in osmoregulation by taking up diverse compatible solutes (Roesser & Müller, 2001 ▸). A common feature of these uptake systems is the presence of a substrate-binding protein (SBP) that is involved in initial recognition of the substrate and thus is responsible for the substrate specificity of the transporter (Du *et al.*, 2011 ▸; Oswald *et al.*, 2008 ▸). Upon binding to a specific substrate, the SBP usually undergoes an open-to-closed conformational change and docks onto the transmembrane subunit to release the substrate for transport across the membrane (Davidson *et al.*, 2008 ▸).

In bacterial pathogens, transporters of compatible solutes have been characterized to play important roles in host infection. In *Escherichia coli*, it was identified that the proline/betaine transporter ProP is involved in colonization of mouse bladder (Bayer *et al.*, 1999 ▸; Culham *et al.*, 1998 ▸). In *Mycobacterium tuberculosis*, which can proliferate by intracellular growth, the ABC transporter ProXVWZ was reported to import glycine betaine from host macrophages to maintain osmotic balance, and deletion of the *proXVWZ* operon led to impairment of initial survival and intracellular growth (Price *et al.*, 2008 ▸). Within the *proXVWZ* operon, the *proX* gene encodes a putative substrate-binding protein, and a *BLAST* search against the PDB revealed that the *M. tuberculosis* ProX (MtProX) protein shares the highest sequence similarity (43%) with the YehZ protein from *Brucella abortus* (Herrou *et al.*, 2017 ▸). However, previous studies indicated a very weak binding affinity (in the millimolar range) between YehZ and glycine betaine, and no interaction was detected between YehZ and other compatible solutes such as proline, choline, ectoine or carnitine (Herrou *et al.*, 2017 ▸; Lang *et al.*, 2015 ▸). Through sequence alignment, we found that MtProX also has very different ligand-binding residues compared with other quaternary ammonium osmoprotectant-binding proteins, implying that MtProX may bind to ligands that are as yet uncharacterized. In this work, we report the production of MtProX and the characterization of binding ligands, together with its crystallization and preliminary X-ray diffraction data.

## 2. Materials and methods   

### 2.1. Macromolecule production   

The gene encoding the MtProX protein was synthesized (GENEWIZ) and cloned into a pET-28b-derived vector with an N-terminal 6×His tag. The recombinant plasmid was transformed into *E. coli* BL21 (DE3) cells (Novagen). The cells were grown to an OD_600 nm_ of 0.6–0.8 at 37°C, and the expression of recombinant protein was induced by 0.2 m*M* isopropyl β-d-1-thiogalactopyranoside for 15 h at 16°C. The cells were collected by centrifugation and resuspended in 20 m*M* Tris–HCl pH 8.0, 150 m*M* NaCl. After 30 min of sonication and centrifugation at 12 000*g* for 25 min, the supernatant containing the soluble protein was collected and loaded onto Ni^2+^–nitrilotriacetate affinity resin (Ni–NTA, Qiagen) equilibrated with 20 m*M* Tris–HCl pH 8.0, 150 m*M* NaCl. The MtProX protein was eluted with 20 m*M* Tris–HCl pH 8.0, 150 m*M* NaCl, 250 m*M* imidazole and further purified by gel filtration on a HiLoad 16/60 Superdex 75 column (GE Healthcare) equilibrated with 20 m*M* Tris–HCl pH 8.0, 150 m*M* NaCl. The peak fractions were collected and concentrated to 30 mg ml^−1^ for crystallization. The protein sample was stored at −80°C and the details of MtProX production are summarized in Table 1 ▸.

### 2.2. Intrinsic fluorescence-quenching assay   

The interaction between MtProX and various compounds was determined by tryptophan fluorescence titration as described previously (Wu *et al.*, 2016 ▸). Several different concentrations of compounds and 2 µ*M* MtProX were prepared in Tris buffer pH 8.0. The fluorescence quenching was conducted in a cuvette with a 1 cm path-length cell. Intrinsic fluorescence spectra of MtProX were recorded using a fluorescence spectrophotometer (Perkin Elmer, California, USA) at room temperature with excitation at 280 nm and emission between 300 and 400 nm. Tryptophan fluorescence spectra were collected before and after titration with different concentrations of compounds. We confirmed that titrating the buffer with the tested compounds produced negligible fluorescence changes under the same experimental conditions. The dissociation constants of the compounds and MtProX were determined by fitting the normalized fluorescence intensity at 340 nm.

### 2.3. Crystallization, data collection and processing   

Crystals of MtProX were grown using the hanging-drop vapour-diffusion method at 16°C by mixing 1 µl 30 mg ml^−1^ protein sample with an equal volume of reservoir solution consisting of 0.2 *M* NaCl, 0.1 *M* Tris pH 8.5, 25%(*w*/*v*) polyethylene glycol 6000. Crystals appeared in two weeks and grew to full size within one month. The details of crystallization are summarized in Table 2 ▸. The crystals were soaked in cryoprotectant (reservoir solution supplemented with 30% glycerol) and flash-cooled in liquid nitrogen. X-ray data were collected at 100 K in a liquid-nitrogen stream using an IµS 3.0 microfocus source with a PHOTON II detector (Bruker) at Lanzhou University. All diffraction data were indexed, integrated and scaled with *HKL*-2000 (Otwinowski & Minor, 1997 ▸). Data-collection statistics are summarized in Table 3 ▸.

## 3. Results and discussion   

**Figure 1:**
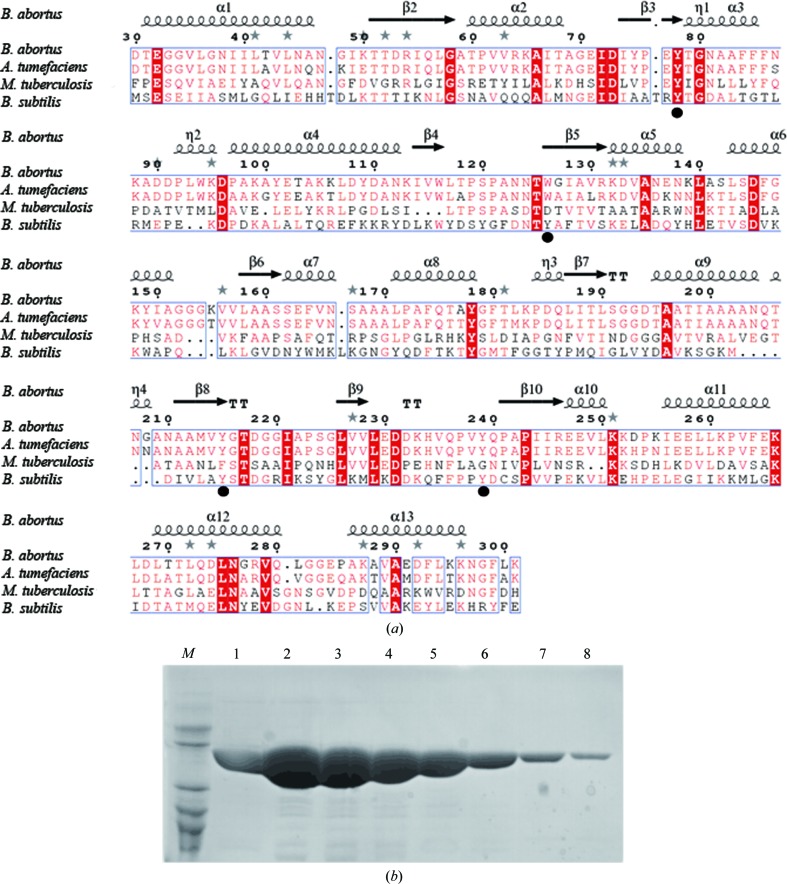
(*a*) Sequence alignment of MtProX homologues, including YehZ from *B. abortus*, ProX from *Agrobacterium tumefaciens* and OpuBC from *Bacillus subtilis*. The aromatic residues that are essential to accommodate the trimethylammonium head moiety of compatible solute molecules in OpuBC are marked by black filled circles below the alignment, and the secondary structure of YehZ is displayed above the alignment. (*b*) 15% SDS–PAGE analysis of MtProX. Lane *M*, low-molecular-weight marker; lanes 1–8, purified recombinant MtProX.

**Figure 2:**
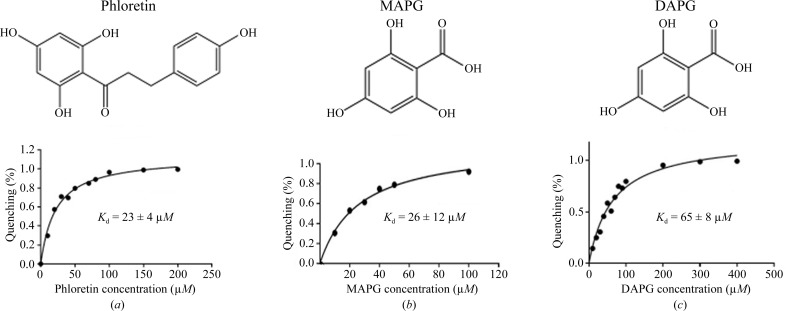
Intrinsic fluorescence titration curves of (*a*) phloretin, (*b*) MAPG and (*c*) DAPG. The dissociation constants are indicated on the plots.

Sequence comparison of MtProX with those of previously solved homologous structures revealed that MtProX does not possess the conserved aromatic residues that are essential to accommodate the trimethylammonium head moiety of compatible solute molecules (Fig. 1 ▸ *a*). To investigate its binding ligands *in vitro*, MtProX was expressed in *E. coli* BL21 with an N-terminal 6×His tag to facilitate affinity purification. After gel filtration, the purity of MtProX was checked by SDS–PAGE (Fig. 1 ▸ *b*). Using a fluorescence-quenching assay, we found that no interaction was detected between MtProX and betaine, choline or carnitine (data not shown). Polyphenols are important secondary metabolites that are mainly produced by plants and can be used as a sole carbon source by certain bacteria (Gorny *et al.*, 1992 ▸). Using *BLAST* (Altschul *et al.*, 1990 ▸), we found an *M. tuberculosis* gene cluster (Rv1714–Rv1716) that shares ∼40% sequence identity with the gene cluster characterized to be involved in polyphenol degradation in *Clostridium* sp. (NCBI reference sequences WP_021630531.1, WP_007491232.1 and WP_021630532.1; Conradt *et al.*, 2016 ▸), suggesting that *M. tuberculosis* may be capable of metabolizing certain polyphenols. Indeed, we found that polyphenols such as phloretin, monoacetylphloro­glucinol (MAPG) and 2,4-dihydroxyacetophloro­glucinol (DAPG) could bind to MtProX with dissociation constants of between 20 and 70 µ*M* (Fig. 2 ▸). Although the physiological ligands of MtProX are currently unknown, our data suggest that MtProX might be involved in the import of polyphenols or analogous compounds in the living environment of *M. tuberculosis*.

**Figure 3:**
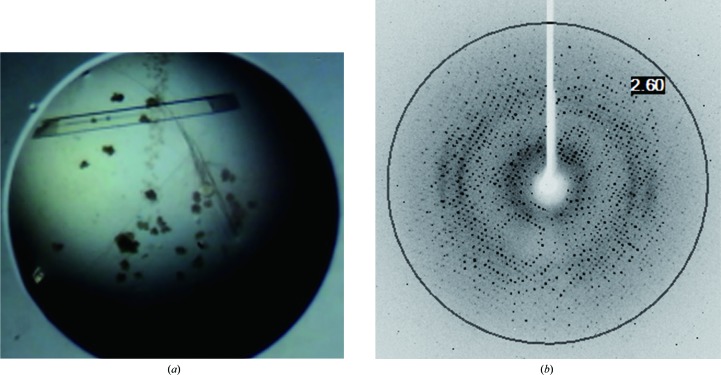
(*a*) A diffraction-quality crystal of MtProX with dimensions of 0.45 × 0.05 × 0.05 mm obtained using the hanging-drop vapour-diffusion method. (*b*) An X-­ray diffraction pattern of an MtProX crystal. The black circle indicates the resolution ring at 2.60 Å.

To gain structural insight into the ligand-binding mechanism of MtProX, we also crystallized and obtained high-quality protein crystals of MtProX. The crystals of MtProX were obtained using a precipitant consisting of 0.2 *M* NaCl, 0.1 *M* Tris pH 8.5, 25%(*w*/*v*) polyethylene glycol 3350 (Fig. 3 ▸ *a*). A complete diffraction data set was collected to 2.10 Å resolution (Fig. 3 ▸ *b*) and the data-collection statistics are listed in Table 1 ▸. The crystal belonged to space group *P*4_3_2_1_2, with unit-cell parameters *a* = *b* = 90.17, *c* = 161.92 Å, α = β = γ = 90°. Assuming the presence of two MtProX molecules in the asymmetric unit, the Matthews coefficient was calculated to be 2.74 Å^3^ Da^−1^, which corresponds to a solvent content of 55%. The structure solution was found by the molecular replacement method with *MOLREP* (Vagin & Teplyakov, 2010 ▸), using the structure of YehZ from *B. abortus* (PDB entry 5teu; 43% sequence similarity) as the search model. The initial *R* factor and *R*_free_ were 42.1 and 46.9%, respectively. Structure refinement is in progress.

## Figures and Tables

**Table 1:** Macromolecule-production information

Source organism	*M. tuberculosis* H37Rv
Expression vector	pET-28b-derived vector
Expression host	*E. coli* strain BL21 (DE3)
Complete amino-acid sequence of the construct produced	MGHHHHHHMSATGSVKSIVVGSGDFPESQVIAEIYAQVLQANGFDVGRRLGIGSRETYILALKDHSIDLVPEYIGNLLLYFQPDATVTMLDAVELELYKRLPGDLSILTPSPASDTDTVTVTAATAARWNLKTIADLAPHSADVKFAAPSAFQTRPSGLPGLRHKYSLDIAPGNFVTINDGGGAVTVRALVEGTATAANLFSTSAAIPQNHLVVLEDPEHNFLAGNIVPLVNSRKKSDHLKDVLDAVSAKLTTAGLAELNAAVSGNSGVDPDQAARKWVRDNGFDHPVRQ

**Table 2:** Crystallization of MtProX

Method	Hanging-drop vapour diffusion
Plate type	16-well plate
Temperature (K)	289
Protein concentration (mg ml^−1^)	30
Buffer composition of protein solution	20 m*M* Tris pH 8.0, 150 m*M* NaCl
Composition of reservoir solution	0.2 *M* NaCl, 0.1 *M* Tris pH 8.5, 25% PEG 3350
Volume and ratio of drop	1 µl protein solution:1 µl reservoir solution
Volume of reservoir (µl)	400

**Table 3:** Data collection and processing Values in parentheses are for the outer shell.

Diffraction source	IµS 3.0 microfocus source
Wavelength (Å)	1.54184
Temperature (K)	100
Detector	PHOTON II
Crystal-to-detector distance (mm)	70
Rotation range per image (°)	1
Total rotation range (°)	360
Exposure time per image (s)	240
Space group	*P*4_3_2_1_2
*a*, *b*, *c* (Å)	90.17, 90.17, 161.92
α, β, γ (°)	90, 90, 90
Mosaicity (°)	0.3
Resolution range (Å)	63.73–2.10 (2.14–2.10)
Total No. of reflections	874316 (17778)
No. of unique reflections	39207 (2142)
Completeness (%)	98.2 (93.4)
Multiplicity	22.3 (8.3)
〈*I*/σ(*I*)〉	17.8 (3.5)
*R*_r.i.m._ †	0.079 (0.326)
Overall *B* factor from Wilson plot (Å^2^)	19.2

† The redundancy-independent merging *R* factor *R*_r.i.m._ was estimated by multiplying the conventional *R*_merge_ value by the factor [*N*/(*N* − 1)]^1/2^, where *N* is the data multiplicity.
